# Investigating the distribution of a rare Colombo-Venezuelan kissing bug, *Rhodnius neivai*, Lent, 1953, using geographical information system-based analyses

**DOI:** 10.1590/0074-02760240106

**Published:** 2024-11-11

**Authors:** Guilherme Sanches Corrêa-do-Nascimento, Cleber Galvão, Gustavo Rocha Leite

**Affiliations:** 1 Universidade Federal do Espírito Santo, Programa de Pós-Graduação em Ciências Biológicas, Vitória, ES, Brasil; 2 Instituto Nacional da Mata Atlântica, Santa Teresa, ES, Brasil; 3 Fundação Oswaldo Cruz-Fiocruz, Laboratório Nacional e Internacional de Referência em Taxonomia de Triatomíneos, Rio de Janeiro, RJ, Brasil; 4 Universidade Federal do Espírito Santo, Departamento de Patologia, Vitória, ES, Brasil

**Keywords:** Rhodnius neivai, Rhodniini, ecological niche model, Chagas disease, neotropics, South America

## Abstract

**BACKGROUND:**

*Rhodnius neivai*, a kissing bug found in the dry regions of Colombia and Venezuela, has limited documented occurrences. While it is not deemed a significant vector for Chagas disease, distributional and ecological studies are essential in monitoring species domiciliation and shedding light on the evolutionary aspects of the Rhodniini tribe.

**OBJECTIVES:**

The study aims to provide a detailed revision of *R. neivai* distribution and evaluate general spatial data quality for ecological niche modelling (ENM). It will also provide the first published ENM for the species, which may aid species sampling and future analytical improvement.

**METHODS:**

Registers and other spatial information were gathered by literature review; data georeferencing, preliminary geographical investigations, and model editing were conducted in GIS platforms; ENMs were built using R and explored the uncertainty of parameters and algorithms.

**FINDINGS:**

Twenty four unique sites were identified, unearthing 17 previously uncovered records. Data lacks robust spatial and temporal precision; however, ENMs had acceptable validations. The models present some variation in suitability but with objective areas for sampling effort.

**MAIN CONCLUSIONS:**

*Rhodnius neivai* distribution is better explained by conditions that characterise dry ecotypes, but further sampling is essential to improve modelling and advance with ecological and evolutive matters.

Triatomines are insects that belong to the Triatominae subfamily (Hemiptera: Reduviidae), known by their blood-sucking habit and to be the vectors of *Trypanosoma cruzi* Chagas, 1909, the causative agent of Chagas disease.^1^ *Rhodnius neivai* Lent, 1953 is a species within the Rhodniini tribe, composed of 23 species grouped in the *Rhodnius* Stål, 1859, and *Psammolestes* Bergroth, 1911, genera, being the second most diverse of the Triatominae tribes.^2^ ^,^ ^3^ ^,^ ^4^ ^,^ ^5^ *R. neivai* has been found in Venezuela and Colombia,^6^ ^,^ ^7^ where kissing bugs are commonly known by the local population as *pito* or *chinche picuda* and *chipo*, respectively.^1^

Most observations of *R. neivai* have occurred in dry ecotypes,^8^ with register in palms, trunks of dead trees, and inside and around domiciles;^9^ ^,^ ^10^ however, there is no evidence to suggest that colonies are maintained in houses.^11^ While *R. neivai* sylvatic habitat and low resistance to starvation suggest the species’ preference for non-human feeding sources and easier epidemiological control, there is a recognised need for surveillance due to the species’ natural *T. cruzi* infection, the capacity to feed on humans, birds and rodents’ blood, aggressive behavior and to its potential for domiciliation.^6^ ^,^ ^12^

*Rhodnius prolixus* has historically been the most relevant vector of Chagas in Colombia and Venezuela.^13^ However, outbreaks of the disease in the acute stage have more recently been associated with oral transmission and with the increased relevance of secondary vectors in the region.^14^ ^,^ ^15^ *R. neivai* findings were not reported in these surveys, but further knowledge of vector biology may be a cautious measure for new epidemiological scenarios.

Spatial data and geographical information system (GIS)-based analyses have played an essential role in epidemiological surveillance efforts, including those for vector-borne diseases.^16^ ^,^ ^17^ In recent years, ecological niche models (ENMs) have gained prominence in studying triatomines in public health^18^ ^,^ ^19^ ^,^ ^20^ and for historical biogeographical research.^21^ ^,^ ^22^

Limited species records^23^ ^,^ ^24^ and poor geographical precision^25^ ^,^ ^26^ often constrain the development of robust ENMs. This is particularly true for kissing bugs that inhabit sylvatic areas and poses a lower risk in the *T. cruzi* epidemiological cycle involving humans; these species generally suffer from insufficient distributional data and heightened spatial sampling bias.^27^ Such is the case for *R. neivai* - few occurrence points are available, both in works with geopolitical reviews of triatomine distribution^6^ ^,^ ^7^ ^,^ ^28^ and the database of American triatomine species occurrence - DataTri.^29^ ^,^ ^30^

New geographical finds for rare species are essential for overcoming macroscale biodiversity shortfalls, as information gaps are based on species distribution (Wallecean shortfall) and evolutive relations (Darwinian shortfall).^31^ Species limit samples are a recognised barrier for evolutive and biogeographical studies within Triatominae.^3^ Including further data in the phylogenetical analysis has provided new taxonomical relations for Reduviidae,^5^ which corroborates the relevance of applying efforts to find rare species. For biogeographical matters, *R. neivai* new findings may be relevant in the exploration of historical connectivity between its dry occurrence region and the Atlantic Forest^22^ to test the niche conservationism hypothesis and distributional range shift due to climate change.

In this study, we review and update the distributional information available in the scientific literature for *R. neivai*, supplementing it with additional georeferenced records for the species. While the data we obtained were temporally biased and suffered from low geographical precision, they were sufficient to produce the species’ first published ENMs. A model identified Annual Precipitation as the most critical predictive variable for explaining the species distribution and provides a foundation for guiding future sampling efforts in known distributional areas.

## MATERIALS AND METHODS

*Occurrence data gathering and georeferencing* - We revised the spatial information for *R. neivai* from older literature in BibTri version 3.0 (Centro de Estudios Parasitológicos y de Vectores - CEPAVE)^32^ and in specialists’ libraries to more recent online publications. We also checked DataTri records^29^ ^,^ ^30^ available in the Global Biodiversity Information Facility (GBIF)^33^ for data incorporation in our analyses and discussion. For georeferencing species records, we used the centroids of the most precise correspondent geopolitical units available in maps made available by the United Nations Office for Coordination of Humanitarian Affairs (UNOCHA).^34^ Register based only on available published maps were georeferenced, overlapping the maps with species occurrence indication and known geographical information. If the geopolitical unit, at least at the municipality level, was unavailable in the previously cited formats, we used the coordinates from Google Maps (https://www.google.com/maps). Geoprocessing used the World Geodetic System 1984 (WGS84) datum in ArcMap V.10.8.^35^

Georeferenced *R. neivai* occurrence data was plotted over the available grid of biogeography provinces built by combining climatic, geological, and biotic criteria with areas of endemism.^36^ We also plotted the register on top of widely accepted ecoregions proposed for the world, claiming to reflect better species and communities’ distribution of biophysical features.^37^ These units may not represent the precise distribution of the *R. neivai* in their delimitations but can provide hints on geographical characteristics and areas of relevance for the species.

**Fig. 1:**
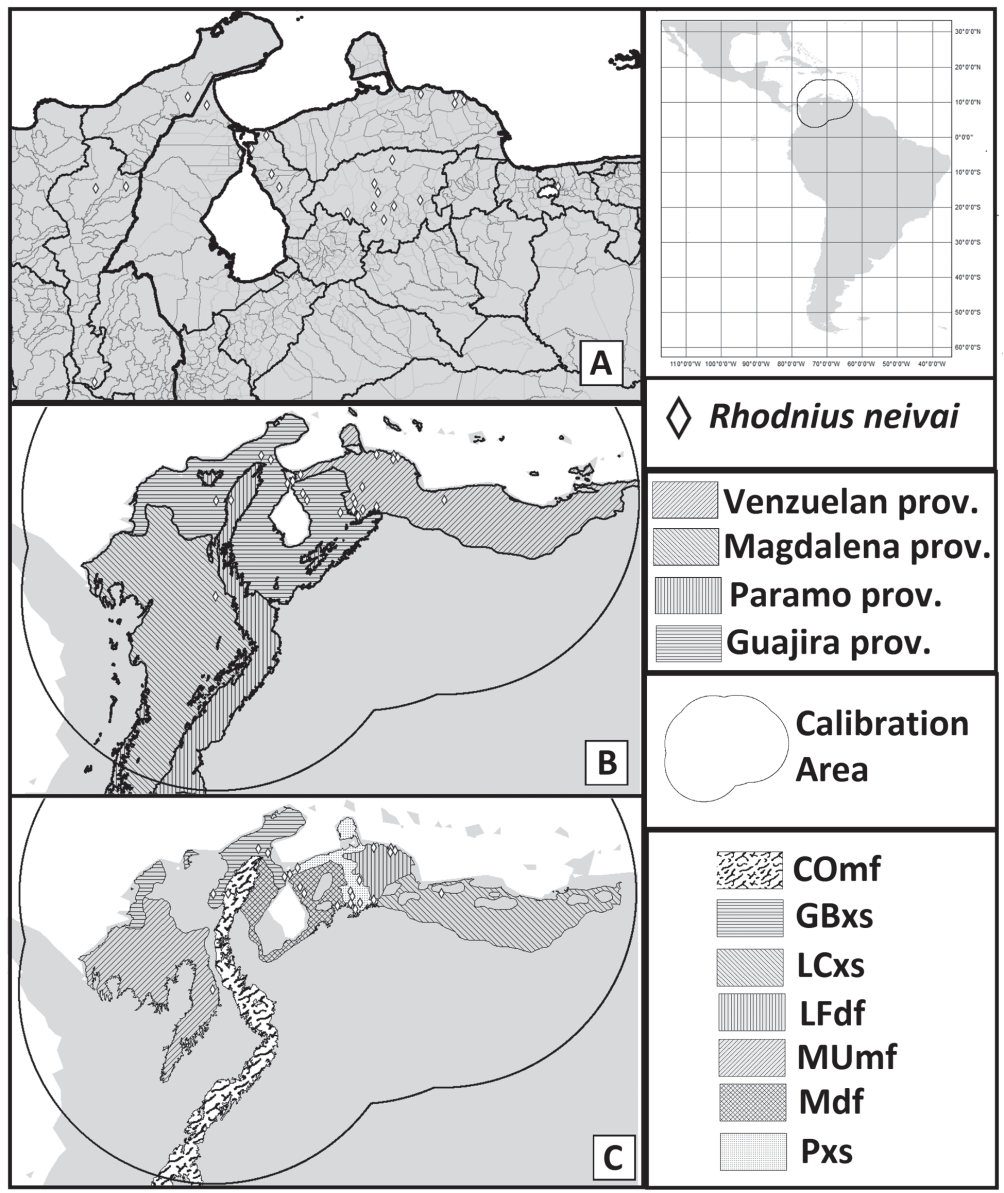
maps of *Rhodnius neivai* geographical occurrences and related geographical relevant areas; register for the *R. neivai* are indicated as a lozenge, the figure in the right upper corner indicates the neotropical region and surrounding areas, the circular area indicates the calibration area used in ecological niche modeling. (A) Geopolitical delimitations of Colombia and Venezuela, where the species has been found, darker and thicker lines indicate higher-level political administrations and clear and thinner lines indicate lower-level administrations. (B) Biogeographical provinces^36^ related to species occurrences indicated by different stripes angulations: Venezuelan (45 degrees); Magdalemo (-45 degrees); Paramo (90 degrees); Guajira (180 degrees). (C) Ecoregions^37^ related to species occurrences indicated by acronyms and different textures: Cordillera Oriental montane forests (COmf - “cowhide” pattern); Guajira-Barranquilla xeric scrub (GBxs - 180-degree stripes); La Costa xeric shrublands (LCxs - -45 degree stripes); Lara-Falcón dry forests (LFdf - 90-degree stripes); Magdalena-Urabá moist forests (MUmf - 45-degree stripes); Maracaibo dry forests (Mdf - checkered pattern); Paraguana xeric scrub (Pxs - dots and white background).

*Ecological niche models* - ENMs were first built following a similar combination approach and the same environmental data previously applied for *Rhodnius domesticus* Neiva and Pinto, 1923 in its calibration area:^22^ We test the same 17 bioclimatic environmental layers in the resolutions of 5 arc minutes from Climatologies at high resolution for the Earth’s land surface areas (CHELSA) in variables choice, available at PaleoClim.^38^ ^,^ ^39^ ^,^ ^40^ The bioclimatic environmental variables Precipitation of Coldest Quarter (Bio 19), Precipitation of Warmest Quarter (Bio 18), Mean Temperature of Driest Quarter (Bio 9), and Mean Temperature of Wettest Quarter (Bio 8) were indicated to present distortions in particular study areas and have been discarded in previous ENMs.^41^ ^,^ ^42^ ^,^ ^43^ ^,^ ^44^ As a priori all 19 variables could be equally relevant for ENMs, we exclude the ones with observed odd discontinued spatial distribution.^45^ Only Bio 18 and Bio 19 were deleted since no other variable presented the spatial artifact. See Supplementary data (Text, Fig. 1) for information on further variables.

ENMs were produced using Maxent version 3.4 in R.^46^ ^,^ ^47^ The calibration area was defined by a five-decimal degree buffer around occurrence points,^22^ ^,^ ^48^ and we used the KUENM package^49^ for the combination of predictive variables and Maxent parameters, models building, and evaluations. We built candidate models based on 70% occurrence data and 30% testing, three predictor variables (from all the 17 possible ones), with highly correlated sets of variables being excluded (|r| > 0.8) to deal with collinearity issues^50^ and data processing limitation.^22^ ^,^ ^51^ The correlation was calculated using the SDM toolbox^52^ in ArcMap V.10.8.^34^ We applied the linear, quadratic, and product feature classes and the following regularisation multipliers, setting applied in exhausting modelling approaches:^53^ 0.1, 0.2, 0.3, 0.4, 0.5, 0.6, 0.7, 0.8, 0.9, 1, 2, 3, 4, 5, 6, 8, 10.

Candidate models were evaluated by statistical significance using partial receiver-operating characteristic curve (ROC), with applied omission-based parameter *E* used in ROC curve delimitations (*E* = 20%; 500 iterations and 50% of data for bootstrapping) and Akaike’s information criterion (AICc) criteria.^49^ ^,^ ^54^ Omission by the corresponding threshold for *E* was not used in the evaluation, given the low amount of occurrence data and a single run of training and testing sampling used in candidate model building. Since we have a low number of records, any single absence in testing omission would be highly relevant, and distinct sampling for training and testing may select distinct ENMs. We choose the set of variables and parameters from the best candidate model according to AICc criteria and produce the final ENM using cross-validation for the sampling test with replications equal to the number of occurrences and background points equal to the number of pixels in the calibration area. The final ENM was the average ENM related to the training samples. Despite the valid critical points made regarding AUC in model evaluation,^55^ we also indicated the mean values from the Maxent output to inform a more general trend of validation in our relatively concise study area.

From the final average model, predictive variables percentage contribution (PC) and permutation importance (PI) in ENM were evaluated to indicate the climatic conditions that better explain species distribution. We also create a categorical model summing two binary ENMs based on the 10-percentile and minimum training presence. As the less restrictive threshold comprehends all pixels of the more restrictive one, the final model indicates three areas: absence of suitability, suitable areas with a higher risk of commission error, and more restrictive areas for species suitability. This model allows objective regions for species sampling efforts to consider two distinct levels of exploration: areas more likely to find the species and more unusual areas where species may occur.

To address the matters of different algorithm responses^56^ and to incorporate Principal Component Analysis (PCA) as an alternative approach to deal with predictive variables collinearity,^57^ a second set of models was produced using the same 17 spatial delimited variables and the following algorithms: Bioclim, Generalises Additive Model, Generalised Linear Model, Maxent and Random Forest. Background points were randomly generated, and model evaluation was conducted by AUC, Kappa, TSS, Jaccard, and Sorensen using the K-Fold (K = 4) data partition method based on the Least Presence Threshold. Models presenting final scores under 0.7 were not taken into consideration. Binary models and omission rates were calculated using the same threshold. For those models, all processing steps were taken using the ENMTML package in R.^58^ The final average, the sum of binary models, and standard deviation models were produced in ArcMap V.10.8 raster calculator.^35^

*Ethics* - The present work was based entirely on secondary triatomine (invertebrate organism) occurrence data gathered by literature review and open data sets, compiled according to all regulations.

## RESULTS

*Occurrence data gathering and georeferencing* - Our revision found 24 registered sites for *R. neivai* in Venezuela and Colombia (Table, Fig. 1A). Only six records indicated the sampling date; none provided precise geographic coordinates. Our list of occurrence sites includes the seven correspondent registers available in DataTri^29^ ^,^ ^30^ and adds 17 uncovered registers from older Venezuelan literature.^9^ ^,^ ^59^ ^,^ ^60^ ^,^ ^61^ ^,^ ^62^ ^,^ ^63^ Some distinct nominal geopolitical information and coordinates differ between the cited literature centroids^28^ ^,^ ^64^ and DataTri [Supplementary data (Table I)]. The divergence of spatial data must be related to distinct georeferencing and spatial geopolitical indication strategies and possible further information provided by specialists to DataTri curators.^29^ ^,^ ^30^

All sites were used in the ENM and overlapping analyses except for the record related to the Sierra Nevada de Santa Marta, which we could not georeferenced given the lack of geopolitical unit indication. Coordinates for the datum seem to be available in DataTri; however, they do not match the geopolitical information indicated in the species literature.^7^ ^,^ ^28^ We prefer not to provide centroids for a larger mountainous area or use coordinates without explicitly confirming their occurrence due to the potential for significant variations of climate conditions in short geographical ranges.^65^

Concerning the areas proposed in Neotropical regionalism,^36^ *R. neivai* records were mainly comprised in regions of Venezuelan and Guajira provinces, with fourteen and seven occurrences, respectively (Table, Fig. 1B). Both provinces of Paramo and Magdalena were related to a single record. For the ecoregions^37^ registers were more common in areas defined by dry conditions (Table, Fig. 1C): Paraguana xeric scrub (10 registers); Guajira-Barranquilla xeric scrub (four registers); Lara-Falcón dry forests (four registers); Maracaibo dry forests (three registers); La Costa xeric shrublands (one register); Cordillera Oriental montane forests (one register); and Magdalena-Urabá moist forests (one register). It is essential to notice that other strategies or more precise information in georeferencing may indicate differences in spatial relations between the records and provinces or ecoregions.

**TABLE:** Compilation of spatial information related to *Rhodnius neivai* geographical distribution

IDR	IDS	Source	S.Date	Country	State	Municipality	Locality	GeoUnit	Latitute	Longitude	Province	Ecoregion
1	1	Lent and Wygodzinsky, 1979	1968	Colombia	Cesar	Valledupar	-	Valledupar	10.2189	-73.4578	Guajira	GBxs
2	2	Morales et al., 1987	1982	Colombia	La Guajira	Maicao	Calabacito	Maicao	11.3816	-72.2950	Guajira	GBxs
3	3	Guhl et al., 2007	<= 2007	Colombia	Cesar	La Paz	-	La Paz	10.2444	-73.0782	Paramo	COmf
4	4	Guhl et al., 2007	<= 2007	Colombia	Cesar	San Alberto	-	San Alberto	7.7698	-73.4722	Magdalena	Mumf
5	1	Guhl et al., 2007	<= 2007	Colombia	Cesar	Valledupar	-	Valledupar	10.2189	-73.4578	Guajira	GBxs
6	5	Guhl et al., 2007	<= 2007	Colombia	Magdalena	-	Sierra Nevada de Santa Marta	-	?	?	?	?
7	6	Lent, 1953	1951	Venezuela	Lara	Camacaro	caserio Parapara	Camacaro	10.2816	-69.9333	Venezuelan	Pxs
8	7	División de Endemias Rurales, 1965	1962-1964	Venezuela	Falcon	-	-	P.MAP	11.4008	-68.8960	Venezuelan	LFdf
9	8	División de Endemias Rurales, 1965	1962-1964	Venezuela	Falcon	-	-	P.MAP	11.2958	-68.9135	Venezuelan	LFdf
10	9	División de Endemias Rurales, 1965	1962-1964	Venezuela	Falcon	-	-	P.MAP	11.3351	-68.7943	Venezuelan	LFdf
11	10	Lent and Juberg, 1969	<= 1996	Venezuela	Lara	Antonio Díaz	caserio Las Playas	Antonio Díaz	9.9852	-69.9367	Venezuelan	Pxs
12	11	Veliz et al., 1972	<= 1972	Venezuela	Lara	Barquisimeto	-	Barquisimeto	10.0677	-69.3473	Venezuelan	Pxs
13	12	Otero et al. 1975ab	<= 1975	Venezuela	Falcon	Puerto Cumarebo	-	Puerto Cumarebo	11.4197	-69.3423	Venezuelan	LFdf
14	13	Otero et al. 1975ab	<= 1975	Venezuela	Lara	Juan Bautista Rodriguez	-	Juan Bautista Rodriguez	9.9964	-69.6844	Venezuelan	Pxs
15	14	Otero et al. 1975ab	<= 1975	Venezuela	Lara	Bolivar	-	Bolivar	9.8235	-69.8073	Venezuelan	Pxs
16	10	Otero et al. 1975ab	<= 1975	Venezuela	Lara	Antonio Díaz	-	Antonio Díaz	9.9852	-69.9367	Venezuelan	Pxs
17	15	Otero et al. 1975ab	<= 1975	Venezuela	Lara	Espinoza de Los Monteros	-	Espinoza de Los Monteros	10.1688	-69.8864	Venezuelan	Pxs
18	16	Otero et al. 1975ab	<= 1975	Venezuela	Lara	Manuel Morillo	-	Manuel Morillo	9.9140	-70.2729	Guajira	Mdf
19	17	Otero et al. 1975ab	<= 1975	Venezuela	Lara	Siquisique	-	Siquisique	10.5798	-69.7056	Venezuelan	Pxs
20	18	Otero et al. 1975ab	<= 1975	Venezuela	Zulia	Cabimas	-	Cabimas	10.4057	-71.2308	Guajira	Mdf
21	19	Otero et al. 1975ab	<= 1975	Venezuela	Zulia	Lagunillas	-	Lagunillas	10.2375	-71.1316	Guajira	Mdf
22	20	Otero et al. 1975ab	<= 1975	Venezuela	Zulia	Altagracia	-	Altagracia	10.7259	-71.4858	Venezuelan	Pxs
23	21	Carcavallo et al. 1976	<= 1976	Venezuela	Zulia	Goajira	-	Goajira	11.2765	-72.0522	Guajira	GBxs
24	22	Carcavallo et al. 1976	<= 1976	Venezuela	Zulia	Faria	-	Faria	10.8897	-71.2989	Venezuelan	Pxs
25	23	de Olaria, 1985	<= 1985	Venezuela	Zulia	Chinquinquirá	sector Delicias	Chinquinquirá	10.6664	-71.6315	Guajira	GBxs
26	24	Harry et al., 2008	<= 2008	Venezuela	Aragua	Maracay	-	Maracay	10.2442	-67.6066	Venezuelan	LCxs
27	24	Pita et al., 2013	<= 2013	Venezuela	Aragua	Maracay	-	Maracay	10.2442	-67.6066	Venezuelan	LCxs

IDR: code for literature; IDS: code for georeferenced site; Source: literature reference; S date: sampling date; GeoUnit: spatial unit georeferenced; Province: biogeographical units;^36^ Ecoregions^37^ names and correspondent acronyms: Cordillera Oriental montane forests [COmf]; Guajira-Barranquilla xeric scrub [GBxs]; La Costa xeric shrublands [LCxs]; Lara-Falcón dry forests [LFdf]; Magdalena-Urabá moist forests [MUmf]; Maracaibo dry forests [Mdf]; Paraguana xeric scrub [Pxs]).

*Ecological niche models* - From our combination of the 17 predictive variables in a set of three and the deletion of sets that contain highly correlated variables (|r| > 0.8 :: correlation matrix available in Supplementary data (Table II), 347 sets of environmental variables remain. A total of 17,697 candidate models were produced from the combination of environmental sets, three feature classes, and 17 regularisation multipliers. The evaluation process indicates 85 statistically significant models following AICc criteria based on 13 selected environmental variables. The model with the best validation was based on a 0.1 regularisation multiplier, quadratic feature class, and the following predictive variables with their correspondent variable contribution (PC) and impact (PI): annual precipitation (Bio 12 :: PC = 86.1; PI = 79.5); precipitation seasonality (Bio 15 :: PC = 10.9; PI = 16); and max temperature of warmest month (Bio 5 :: PC = 3; PI = 4.5).

**Fig. 2:**
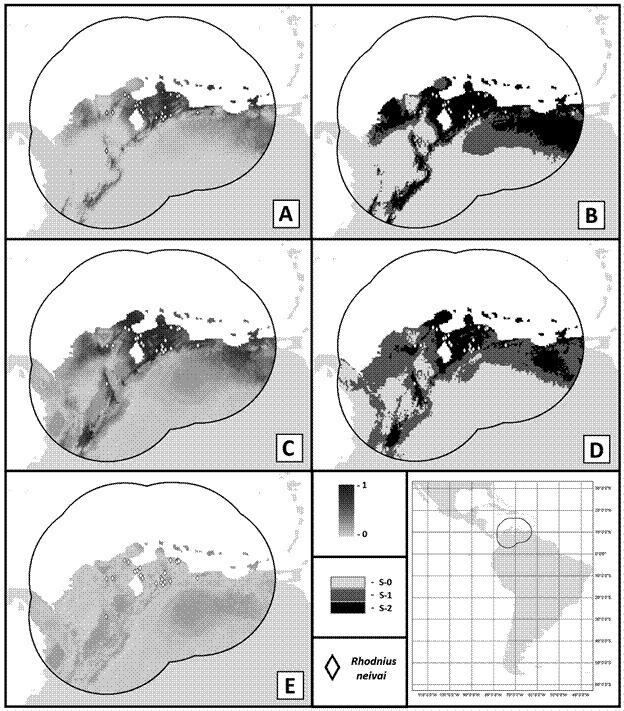
ecological niche models (ENMs) for *Rhodnius neivai*; register for the species are indicated as a lozenge, the figure in the left-down corner indicates the neotropical region and surrounding areas, the circular area indicates the calibration area for the ENMs; note the analyses were only made for this particular geographical region. On the left column are present continuous maps with float values, ranging from zero (lighter gray) to one (darker gray). Maps in the right column are derived from the sum of binary models and present categories: S-0 (no suitability), S-1 (suitability given one scenario), and S-2 (suitability given both scenarios). (A) Three variables ENM. (B) Categorical model based on the sum of binary three variables ENMs, scenarios are based on thresholds. (C) Principal Component Analysis (PCA) variables ENM. (D) Categorical model based on the sum of binary PCA ENMs, with scenarios, based on different algorithms. (E) Standard deviation model for PCA variables ENM, based on algorithm variation.

The final ENM was built using 23 occurrence points; therefore, as previously stipulated, 23 repetitions for cross-validation and background points equals 13,663 pixels, and the test AUC was equal to 0.898. Suitability was higher in dryer northern parts of the study area, with values varying between practically zero to near 0.87 (Fig. 2A). Threshold values for the 10-percentile training presence equals 0.1537, and for the minimum training presence, 0.0319. The categorical model also indicates the most restrictive area for suitable conditions in the north and less restrictive in its surroundings, being more significant in the eastern part of the map (Fig. 2B).

For the alternative model, PCA produced five distinct variables, the model omission rate was always equal to zero, and from further model validation, only Maxent and Random Forest present scores above 0.7 [see Supplementary data (Table III, Fig. 2) for complete model validation and individual ENMs, respectively]:

Maxent - AUC = 0.8042; Kappa = 0.7417; TSS = 0.7417; Jaccard = 0.7976; Sorensen = 0.8866. The continuous model presented a visual aspect closer to the previous one, which was made with only three variables. However, the binary version had a more extensive area indicating species presence [Supplementary data (Fig. 2)].

Random Forest - AUC = 0.8250; Kappa = 0.7417; TSS = 0.7417; Jaccard = 0. 8036; Sorensen = 0.8888. The continuous model visually presents a larger suitability area; however, the values seemed generally lower. The binary model indicates the smallest area for the models [Supplementary data (Fig. 2)].

Both average models presented somewhat similar suitability distribution, with this alternative model possessing more intermediary values (Fig. 2C). In the binary model, a larger suitable region appears west of the study area. However, the divergence between algorithm outputs is larger, making a visually smaller area of consensus (Fig. 2D). The standard deviation model indicates a significant extent with values up to 0.309 of variation, which include areas of high suitability (Fig. 2E).

## DISCUSSION

*Historical sampling for R. neivai* - We provide detailed information on the sampling history for *R. neivai* in this section, given the rare records for the species, starting from its description: *R. neivai* was first collected in 1951 by Suárez in the Locality of caserío Parapara, Camacaro, state of Lara, Venezuela, then was send through Gabaldon to Lent that described the species.^66^ ^,^ ^67^ The type specimen was indicated to be deposited in the entomological collection of the Oswaldo Cruz Institute (CT-IOC) in Rio de Janeiro, Brazil.^64^ ^,^ ^66^

An official Venezuelan campaign against Chagas diseases report acknowledges the occurrence of *R. neivai* in Lara. It indicates that what seems to be three records for *R. neivai* in the state of Falcón were found in houses between 1962 and 1964, but the localities’ or municipalities’ names were not provided. Still, the distribution in the country map was made available.^59^ According to our georeferencing, all points are contained in the geopolitical delimitations of the locality of San José de la Costa, Municipality of Piritu.

A genitalia study of the *Rhodnius* genera indicated an allotype from the locality of caserío Las Playas, Municipality of Antonio Diaz, state of Lara.^68^ The species was also infected with *T. cruzi* in Barquisimeto, state of Lara, Venezuela.^6^ ^,^ ^60^ Later, the work acknowledges the species distribution in Lara and Falcón in a small table with species identification made up to 1959 and 1965, respectively, but does not provide precise geopolitical information and strangely indicates in the main text that *R. neivai* to be only captured once in the state of Lara.^69^

In dry regions of Puerto Cumarebo, Falcón, Venezuela, *Rhodnius brethesi* Matta, 1919 was registered, a triatomine typical of wet Amazon areas; however, taxonomical revision indicated that specimens were *R. neivai*.^61^ ^,^ ^62^ The same authors also made new registers in the state of Zulia: Cabinas; Lagunillas; and Altagracia; and in Lara: Sisquisique; Juan Bautista Rodriguez; Bolivar; Antonio Diáz; Espinoza de Los Monteros; and Manuel Morillo.^61^ A World Health Organization (WHO) report^62^ provided identical records, except for Espinoza de Los Monteros, which was indicated as “E. de los Rios”. No mention of the registers we georeferenced to be in San José de la Costa was made after the campaign report,^59^ making the records somewhat dubious.

Sylvatic field searches for triatomines made in the state of Zulia register *R. neivai* in Faría on a dry tree and in Goajira (georeferenced by us as Guajira) associated with palm.^9^ Actualisation of triatomine distribution according to biogeographical zonation indicates species distribution related to dry regions in Lara, Falcón, and Zulia.^8^ The species was posteriorly found inside a house in el sector Delicias, Chinquinquirá, Maracaibo, in the state of Zulia.^63^ Genetical works for the Rhodniini species also indicate *R. neivai* presence in Maracay, Venezuela, related to specimens deposited in Oswaldo Cruz Institute, Fiocruz Insectary, Rio de Janeiro, Brazil.^70^ ^,^ ^71^ We did not find any further registers for the species in Venezuela; its distribution is related to northwest regions in the country (Fig. 1A).

In Colombia, the first register for *R. neivai* was made in 1968 and found in “Magdalena: Valledupar”;^11^ posteriorly, the site was indicated to be comprised in the state of Cesar.^72^ The second register in the country was made using artificial lights in the corregimiento de Calabacito, Maicao, state of La Guajira, in 1982.^73^ In a review of triatomine distribution in Colombia, new registers were also indicated from the state of Cesar in La Paz, San Alberto, and others for Valledupar and in the state of Magdalena in the Sierra Nevada de Santa Marta.^28^ In fieldwork and database assemblage for triatomines in Colombia, it is indicated that *R. neivai* has not registered in further municipalities since the 2007 published revision for the country.^7^ ^,^ ^28^

The known register of *R. neivai* in Colombia is mainly related to the country’s northern areas (Fig. 1A). The occurrence in San Alberto was by far the most isolated and southern point in all species distribution and is the only one in the biological province of Magdalena and the Magdalena-Urabá moist forests ecoregion. The unusual record may be related to species random dispersion or population related to small available habitats in the region. La Paz’s register is also the single point in Paramo province and Cordillera Oriental montane forests ecoregion. However, this may be related to geospatial imprecision and the narrow dimension of the biogeographical areas.

The record of the Sierra Nevada de Santa Marta is proposed to be comprised in Magdalena.^28^ ^,^ ^30^ Still, the most recent compilation on species distribution in Colombia does not acknowledge any of its municipalities for the record.^7^ It is also possible that changes in names or delimitations of geopolitical units lead to mismatched spatial information, but we did not evaluate such matters. The short spatial variation of biodiversity and climatic zonation found across mountain elevations^65^ ^,^ ^74^ makes high precision on geographical occurrences of *R. neivai* even more critical to understanding the diversity of climate conditions the species can inhabit.

*Ecological niche models* - The latitudinal gradient is an essential factor related to triatomine richness.^75^ Other macroecological analyses include triatomine richness found in dryer areas in the neotropics and the temperature variables being relevant in explaining species richness and distribution, even though other variables may be relevant to explain a large part of diversity patterns.^76^ Our first analyses support the relation of *R. neivai* occurrences in dry environments^10^ but indicate precipitation variables being more relevant in explaining the species distribution. Annual Mean Precipitation contribution and importance were far more relevant than both other two variables combined. This may be explained by the low number of variables used in model building and the low correlation between each other (|r| < 0.8). As further registers are made for the species, more variables may be added to modeling, and more complex environmental responses may be indicated. However, the climate relation is ecologically meaningful, and precipitation variables could remain relevant for future *R. neivai* models, given that precipitation seasonality was also more relevant than the maximum temperature of the warmest month.

ENMs for *Triatoma dimidiata* Latreille, 1811 indicate annual mean precipitation as one of Colombia’s most relevant variables for the triatomine distribution.^77^ ENMs for other triatomines in the country exclude this variable given their strategy of dealing with variables collinearity; it indicates other precipitation variables with high contribution for the models, often precipitation seasonality being of critical relevance^78^ and makes us wonder if precipitation-derived variables may be necessary for distribution in the macro biodiversity scale in the region. For Venezuelan triatomines ENMs, on the other hand, only temperature-derived variables were relevant for the models,^79^ and precipitation variables were considered less critical for *Rhodnius prolixus* Stål, 1859.^80^ Some of these applications used study areas based on geopolitical delimitation; applying the species’ total distribution and meaningful ecological calibration areas may provide distinct results for the models.^81^

ENMs for triatomines using full distribution and calibration areas based on ecoregions indicate that some precipitation variables were relevant for *Triatoma maculata* Erichson, 1848 (with register both in Colombia and Venezuela) and *Rhodnius pallescens* Barber 1932, (with register in Colombia but not in Venezuela), the latter including annual mean precipitation as a significant variable in model building.^51^ The trend of variable responses for particular regions is of general biogeographical interest. However, these areas include different environmental regions and distinct species distributions that may indicate specific variables’ contribution and importance in their corresponding models. For example, distinct variables act as limiting factors based on ENMs for different species that also occur in the general region.^82^

Despite relevant suitability being present in some humid regions of the study area, the higher spatially cluster values for the first model were mainly in northern dryer parts (Fig. 2A). According to our georeferenced data, Southerner and Westerner suitable areas may represent montane valleys or slopes, and species distribution in those points is the most uncertain. Suitability was found around, but not in, the Sierra Nevada de Santa Marta’s general area, which may indicate the record from lower elevation regions. The Westerner points for *R. neivai* are from San Alberto, La Paz, and Valledupar. They are only supported by the most unrestricted threshold (Fig. 2B). Species could have dispersed from near suitable areas, or unprecise georeferencing could influence the response. Centroids are points that provide spatial bias when applied in ENMs.^25^ ^,^ ^26^ Given ecological coherence in variables and the suitability of spatial distribution, we do not trust that these spatial errors may represent a substantial risk in model response. However, we have a small dataset, so they have become highly relevant in model evaluation, especially regarding omission rate.

The alternative model based on distinct algorithms, PCA variables, and a more inclusive threshold indicates a similar response to continuous (Fig. 2C) and binary (Fig. 2D) derived models. Some larger suitable areas are indicated in westerner regions of Colombia (Fig. 2D). However, the binary divergence between Maxent and Random Forest models (Fig. 2D) and some high areas of standard deviation (Fig. 2E) make the areas less robust. Considering maps available for biomes and ecoregions of Colombia,^83^ these newly suitable areas comprise large moist regions that would be less expected for species occurrence. Among the previously discussed biases, this result could be influenced by the more inclusive threshold. As the binary models used PCA variables and the least presence values for threshold, the model will not be highly informative for discussion on the ecological influence of the variable and the occurrence sites that are less robust.

Further sampling for the species with more accurate geospatial information may provide robustness in *R. neivai* ENMs and supply better temporal data, given the old date of the records (Table). However, we acknowledge the substantial limitations of sampling rare sylvatic triatomines.^27^ On the other hand, the effort may prove helpful in exploring interesting biogeographical events in Rhodniini historical biogeography: *R. domesticus*, proposed to be *R. neivai* sister species,^84^ is related to more distant moist regions in the Atlantic Forest.^18^ ^,^ ^22^ ^,^ ^85^ Transferring ENMs for *R. neivai* may help to explore the climatic range shift effects in species distribution and explain the current distance between species. Well-established niches in environmental space may also help test significant niche divergence in cladogenesis. Niche conservationism was proposed to fit better niche relations between sister species of North and Central America triatomines.^86^ However, *R. neivai* and *R. domesticus* likely evolutive “niche dissimilarity” may indicate distinct relations in triatomine and South American groups. We trust that to test the hypothesis in the future, it would be required to make occurrence data for both *R. domesticus* ^22^ and *R. neivai* more robust and certify the phylogenetical relation between the species.

*In conclusion* - As expected, our literature revision indicates that available information on *R. neivai* distribution is scarce and has low spatial and temporal precision. Nevertheless, the 17 recovered records in Venezuela provide a better occurrence sample for applying ecological niche modelling. Our model indicates annual mean precipitation as the predictive variable that significantly influences the explanation of species distribution. The relation makes ecological sense for *R. neivai*, given its historical occurrence in dry environments.^10^ The ENMs have limitations, and some occurrences for the species may be geographically dislocated from their natural source (the Sierra Nevada de Santa Marta, San Alberto, La Paz, and Valledupar). Our models may aid the field search for *R. neivai* in a more practical context. Any new record for the species may provide important data for the application of the method, and future sampling must aim to provide high geographical precision; the coordinates for the register would be the most suitable type of information. More complex ENMs and biogeographical hypotheses may be explored in the future as new registers for the species are made.
